# Post-operative outcomes after cleft palate repair in syndromic and non-syndromic children: a systematic review protocol

**DOI:** 10.1186/s13643-017-0438-2

**Published:** 2017-03-09

**Authors:** Zach Zhang, Michael Stein, Nigel Mercer, Claudia Malic

**Affiliations:** 1 Faculty of Medicine, University of Ottawa, Ottawa, Canada; 2 Division of Plastic Surgery, University of Ottawa, Ottawa, Canada; 3 Cleft Unit, Frenchay Hospital, Bristol, BS16 1LE UK

**Keywords:** Cleft palate, Post-operative complication, Palatal fistula, Velopharyngeal insufficiency, Midface hypoplasia, Facial growth

## Abstract

**Background:**

There is a lack of high-level evidence on the surgical management of cleft palate. An appreciation of the differences in the complication rates between different surgical techniques and timing of repair is essential in optimizing cleft palate management.

**Method:**

A comprehensive electronic database search will be conducted on the complication rates associated with cleft palate repair using MEDLINE, EMBASE, and the Cochrane Central Register of Controlled Trials. Two independent reviewers with expertise in cleft pathology will screen all appropriate titles, abstracts, and full-text publications prior to deciding whether each meet the predetermined inclusion criteria. The study findings will be tabulated and summarized. The primary outcomes will be the rate of palatal fistula, the incidence and severity of velopharyngeal insufficiency, and the rate of maxillary hypoplasia with different techniques and also the timing of the repair. A meta-analysis will be conducted using a random effects model.

**Discussion:**

The evidence behind the optimal surgical approach to cleft palate repair is minimal, with no gold standard technique identified to date for a certain type of cleft palate. It is essential to appreciate how the complication rates differ between each surgical technique and each time point of repair, in order to optimize the management of these patients. A more critical evaluation of the outcomes of different cleft palate repair methods may also provide insight into more effective surgical approaches for different types of cleft palates.

**Electronic supplementary material:**

The online version of this article (doi:10.1186/s13643-017-0438-2) contains supplementary material, which is available to authorized users.

## Background

Cleft palate is the most common craniofacial birth defect, occurring in 1 in 2000 live births [1]. The management of patients with cleft palate pathology is complex and requires a multidisciplinary approach, which includes plastic surgeons, maxillofacial surgeons (cleft surgeons), otolaryngologists, speech/language pathologists, audiologists, dentists, orthodontists, psychologists, geneticists, and social workers. Over the last two decades, significant efforts have been made to both standardize and streamline the perioperative management of patients with cleft palate worldwide with different degrees of success in Europe, the UK, and North America. Despite these efforts, both the surgical repair and perioperative management of these patients vary considerably between centers and ultimately are decided upon by the surgeon’s knowledge, training, and expertise.

The extent of the defect and the complexity of associated factors are as highly variable as the timing of surgery. As a consequence, there is a paucity of high-level evidence [2] outlining a gold standard for the ideal surgical approach to cleft palate repair.

**Fig. 1:**
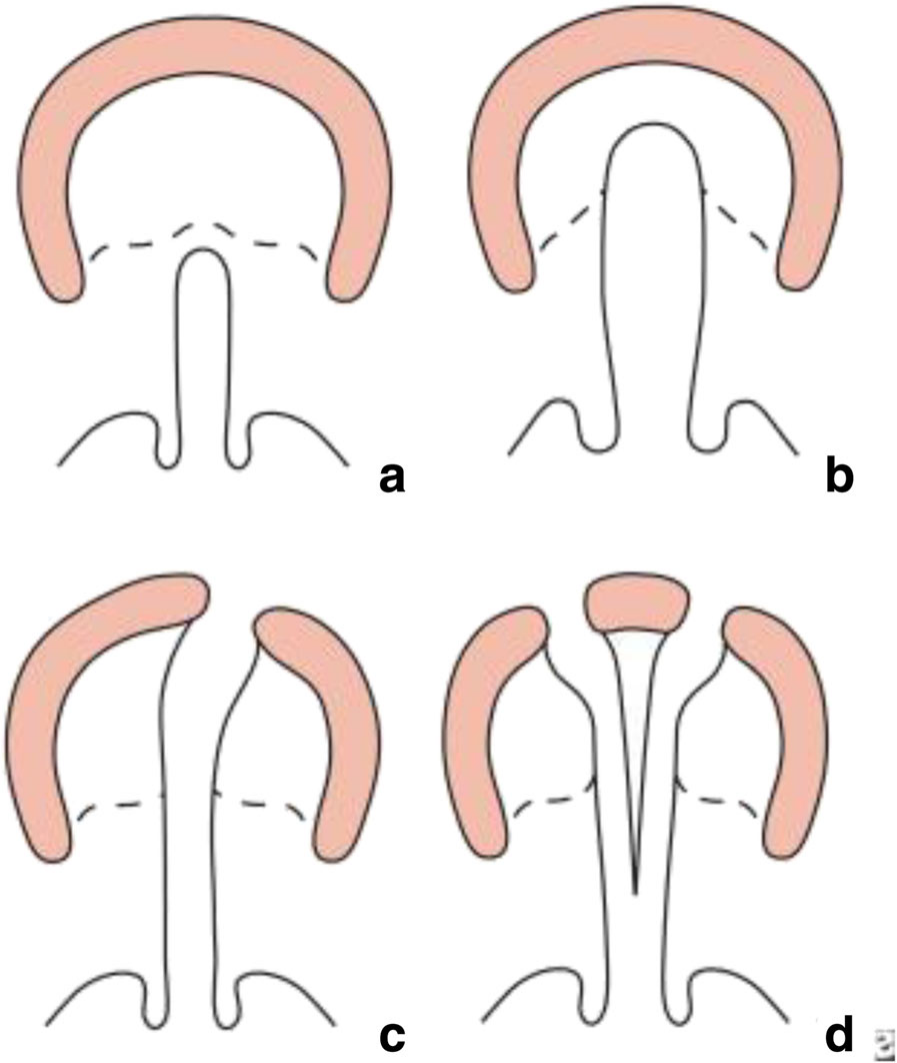
**a**–**d** Veau classification system of cleft lip and palate

**Fig. 2:**
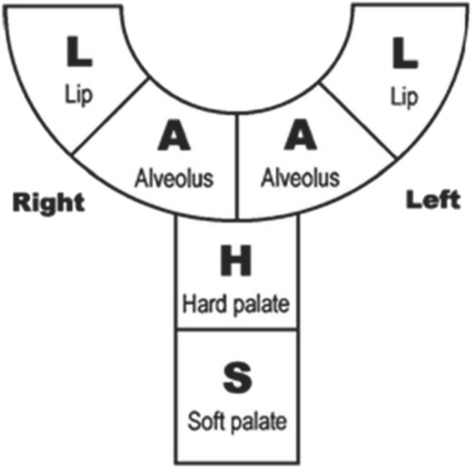
LAHSAL system for the classification of cleft lip and palate

The techniques used for cleft palate repair vary depending on the specific type of cleft palate. In terms of defining the extent of a cleft palate, several classifications are available in the literature. The most frequent one used in both clinical work and research is the Veau classification, which scores cleft palates by the anatomic disruption of the primary and secondary palates (Fig. 1). Veau classes I and II denote clefts of the secondary palate with class I being incomplete and limited to the soft palate and class II involving both the hard and soft palates. Veau classes III and IV denote complete clefts including both the lip and palates with class III being unilateral and class IV being bilateral [3]. In the UK, the LAHSAL code is used because it is much easier to use in practice (Fig. 2) [4, 5].

Surgical repair of cleft palate is associated with three major long-term complications: palatal fistula, velopharyngeal insufficiency (VPI), and midface hypoplasia—which could be considered also as metrics and quality indicators [6, 7]. A 2012 systematic review on protocols in cleft lip and palate treatment found a paucity of literature outlining surgical repair techniques. Furthermore, no post-operative complications were reviewed in this review [8]. It is clear that the lack of evidence-based medicine in cleft palate management warrants further investigation.

In addition to the array of surgical approaches, there is also variability on the optimal timing to perform palate repair [9]. As transverse facial growth is not completed until 5 years of age, some have advocated a later cleft palate repair, even as late as age 8–10, to reduce the risk of midface hypoplasia. There is also evidence to support an earlier repair before the age of 2, as it is argued it improves speech development and results in better integration in society with less psychosocial impact for children and families [10]. Furthermore, some surgeons manage cleft palate repair in two stages (with soft palate repair at 3–6 months and hard palate repair at 15–18 months), while others advocate a single-stage repair with both the soft and hard palates being repaired simultaneously. To date, there are only a few small, cohort, long-term studies on the incidence of midface hypoplasia with each method of cleft palate repair [9]. Furthermore, there is a variety of techniques, as well as adaptations, in order to repair cleft palate in a single stage. Some of these techniques are push-back techniques, unipedicle or bipedicle palatal mucosa flaps, hybrids, or double-opposing Z-plasty for the soft palate. Lastly, intravelar veloplasty (IVVP) was introduced in the 1970s by Kriens and intends to repair the velar muscles across the midline, after careful dissection for its abnormal insertion on the cleft edge [11]. While making intellectual sense, there is little evidence of its efficacy compared with no IVVP.

The objective of this systematic review is to evaluate and appraise the evidence of various cleft palate repair techniques and their timing in both syndromic and non-syndromic patients, with particular attention to complication rates of palatal fistula formation, velopharyngeal insufficiency, and midface growth retardation as indicators of the success/failure of the chosen technique.

## Methods

### Study criteria

#### Participants

Studies to be included will examine patients with Veau class I–IV cleft palates who underwent cleft palate repair surgery and were followed up long enough to appreciate the repair outcome and complication rates. Children with syndromic and non-syndromic clefts will be separated and studied independently.

#### Intervention

The type of surgical cleft palate repair intervention and the timing of the cleft palate repair will be the primary focus of the review. The type of surgical cleft palate repair will include but not be limited to the following: intravelar veloplasty and Furlow double-opposing Z-plasty of the soft palate, Von Langenbeck palate repair, push-back palate repair, Veau-Wardill-Kilner palatoplasty, Bardach two-flap palatoplasty, and the use of vomer flap. The types of intervention will be categorized based on the use of muscle reposition, the vascularity of the mucosal palatal flaps, the method of nasal layer closure, and the timing of the repair.

#### Comparators

Given the broad perspective for interventions of interest, several comparisons will be relevant to include. Some will come from observational designs and others from experimental studies.

Cleft palate repair techniques:

- Intravelar veloplasty
- Furlow double-opposing Z-plasty
- Von Langenbeck palate repair
- Push-back palate repair
- Veau-Wardill-Kilner palatoplasty
- Bardach two-flap palatoplasty
- Vomer flap

Categorization of cleft palate repair techniques:

Muscle reposition is an additional step in the surgical repair of cleft palate. In the Furlow double-opposing Z-plasty, the muscle is part of the flap design. In the other techniques, an intravelar veloplasty is required.

Vascularity of the mucosal palatal flaps:

- Bipedicle
  - ○ Von Langenbeck repair
  - ○ Furlow double-opposing Z-plasty + Von Langenbeck repair
- Unipedicle
  - ○ Veau-Wardill-Kilner palatoplasty
  - ○ Bardach two-flap palatoplasty
  - ○ Push-back palate repair
- Hybrid
  - ○ Half Von Langenbeck + half Veau-Wardill-Kilner palatoplasty

Nasal layer closure:

- Nasal palatal mucosa and addition of vomer flap if the cleft is too wide for direct repair of the nasal mucosa

Cleft palate repair timing:

- Before age of 2 with the following subsets:
  - ○ Simultaneous repair: both the soft and hard palates between 9 and 12 months
  - ○ Two-stage repair
    - ▪ Soft palate repair at 3–6 months
    - ▪ Hard palate repair at 15–18 months
- After the age of 2

#### Outcome measures

The primary interest of this review will be the complications associated with each technique. The following complications will be analyzed: rate of palatal fistula, incidence of VPI requiring surgical intervention, and the rate of midface hypoplasia. VPI assessment is assessor dependent, as there are no strict objective measurements in determining the degree of the VPI. The review will look into the VPI who required surgery and those who do not. Within the subgroup which did not require surgery, we will look into the degree of the VPI if the data is available. The success of the procedures is defined by the normalization of speech, no or minimal midface hypoplasia, and no palatal fistula.

#### Follow-up length

Studies will be selected for inclusion based on the length of follow-up. A minimum of 6 months is required for the evaluation of fistula as an outcome, and at least 2 years is required to determine the incidence of VPI if the repair is performed in the first year of life. With regard to midface hypoplasia, long-term follow-up till adolescence and adulthood is required. In addition, the incidence of any orthognathic surgery will be included in the scope of this study if the data is available.

#### Study design

We will include randomized controlled trials (RCTs), cluster RCTs, non-randomized controlled clinical trials, interrupted time series, and prospective and retrospective comparative cohort studies. Case series and case reports will be excluded.

#### Setting

There will be no restriction by type of setting.

#### Language

We will include articles reported in English and French.

### Search strategy

A comprehensive electronic database search will be conducted using MEDLINE (OVID interface, 1948 onwards), EMBASE (OVID interface, 1980 onwards), and the Cochrane Central Register of Controlled Trials (Wiley interface, current issue) from inception to 2016 by collaborating with healthcare librarians. Key search terms will include “cleft palate,” “palatoplasty,” “fistula,” “velopharyngeal incompetence,” and “facial growth.” The search will be limited to studies in English and French and human studies, and there will be no experimental studies. The search strategy will be peer-reviewed with several librarians. A sample search strategy is shown in Additional file 1.

### Screening

The search results will be uploaded to RefWorks software and de-duplicated. The updated results will then be uploaded to Covidence software for primary screening. Two reviewers will independently screen all titles and abstracts. We will obtain full articles for all titles that appear to meet the inclusion criteria or where there is any uncertainty. The two reviewers will then screen all full-text articles independently, and any areas of disagreement will be resolved by consensus. Neither of the review authors will be blinded to the journal titles or to the study authors or institutions.

### Data extraction

The authors will collect the data independently using a data collection form without duplication. The following data will be collected and added to a standardized data collection form:

- Study design
- Eligibility and exclusion criteria for study participation
- Syndromic vs. non-syndromic
- Cleft palate Veau classification status
- Type of surgical intervention technique
- Timing of the repair
- Procedure complications
  - ○ Rate of palatal fistula
  - ○ Rate of VPI requiring surgical intervention
  - ○ Rate of midface hypoplasia
  - ○ “Normalization” of speech
- Methods of statistical analysis

### Data analysis

The study findings will be tabulated and summarized using the statistical software RevMan 5.1, according to the guideline referenced in the *Cochrane Handbook for Systematic Reviews of Interventions*. The primary outcomes will be the rate of palatal fistula, VPI requiring surgery, and maxillary hypoplasia. Clinical heterogeneity will be tested by considering the variability in participant factors among trials and trial factors using the *χ* ^2^ test and *I* ^2^ statistic. If studies are sufficiently homogeneous in terms of design and comparator, we will conduct a meta-analysis using a random effects model. If the data are appropriate for quantitative synthesis, the rates of complication will be determined by using risk ratio with 95% confidence interval. Since a number of different study designs such as RCT, CCT, ITS, and prospective and retrospective comparative cohort studies will be included in the review, it is likely that heterogeneity will be too high for meta-analysis. Instead, different designs will be separated and homogeneity will be assessed within each group for the possibility of subgroup analysis. We will also be stratifying the findings by the study’s population (syndromic vs. non-syndromic), interventions used for cleft palate repair, context, outcomes (one or more of the three: rate of palatal fistula, VPI, maxillary hypoplasia), and validity. If heterogeneity is deemed too high even within each subgroup, then a systematic narrative synthesis will be provided with information presented in text and tables to summarize and explain the characteristics and findings of the included studies. The narrative synthesis will explore the relationship and findings both within and between the included studies, in line with the guidance from the Centre for Reviews and Dissemination.

To facilitate the assessment of possible risk of bias for each study, we will collect information using the *Cochrane Collaboration Tool for Assessing Risk of Bias*, which covers both study- and outcome-level bias in the domains of selection bias, performance bias, detection bias, attrition bias, reporting bias, and any other we may come across during our review. For selection bias, we will be reviewing the adequate generation of two or more groups of cleft palate patients with similar characteristics. For performance bias, we will be reviewing if the patients and study personnel were blinded to the intervention; however, this is unlikely given the intervention involves surgery. We will be assessing detection bias based on whether the assessor for the outcome was blinded to the knowledge of the type and timing of the repair the cleft patient received. For attrition and exclusion bias, we will assess for the completeness of outcome data for each outcome. If there are missing data, we will state the number of outcome assessed compared with the total number of participants that received interventions and if any reason of attrition/exclusion was reported. These judgments will be made independently by two review authors based on the criteria for judging the risk of bias (Table 8.5.c in the *Cochrane Handbook*) [12]. Disagreements will be resolved by discussion.

### Data synthesis

If studies are sufficiently homogeneous in terms of design and comparator, we will conduct meta-analyses using a random effects model. We will test the clinical heterogeneity by considering the variability in participant factors among trials (for example, age) and trial factors (randomization concealment, blinding of outcome assessment, losses to follow-up, treatment type, co-interventions). Statistical heterogeneity will be tested using the *χ* ^2^ test (significance level, 0.1) and *I* ^2^ statistic (0 to 40%, might not be important; 30 to 60%, may represent moderate heterogeneity; 50 to 90%, may represent substantial heterogeneity; 75 to 100%, considerable heterogeneity). If high levels of heterogeneity among the trials exist (*I* ^2^ ≥ 50% or *P* < 0.1), the study design and characteristics in the included studies will be analyzed. We will try to explain the source of heterogeneity by subgroup analysis or sensitivity analysis. Subgroup analysis will consist of patient characteristics (sex, age, syndromic vs. non-syndromic, Veau classification, and geographic location of the repair), study design, and length of follow-up. Sensitivity analysis will be performed in categories of quality components (full-text publications vs. abstracts, preliminary results vs. mature results, published vs. unpublished data) and risk of bias.

For dichotomous data (occurrence of palatal fistula, VPI requiring surgical intervention, maxillary hypoplasia), measurement of treatment effect will be determined by using risk ratio (RR) with 95% confidence interval (CI). None of the studied outcome data is continuous. Skewed data and non-quantitative data will be presented descriptively.

The primary analysis will be per individual randomized; however, all included trials will be assessed in order to determine the unit of randomization. The level at which randomization occurred and the match between the number of observations and randomized units will be evaluated. Special issues in the analysis of studies with non-standard design, like cluster randomized trials, crossover trials, and studies with multiple treatment groups, will be addressed. For cluster randomized trials, we will extract an interclass correlation co-efficient to modify the results according to the methods described in the *Cochrane Handbook for Systematic Reviews of Interventions* [12]. For crossover trials, a major concern is the carry-over effect. We will only use the data from the first phase, guided by the Cochrane Heart Group. When a study has more than two treatment groups, we will present the additional treatment arms. Where the additional treatment arms are not relevant, they will not be taken into account.

When there are missing data, we will attempt to contact the original authors of the study to obtain the relevant missing data. Important numerical data will be carefully evaluated. If missing data cannot be obtained, an imputation method will be used. We will use sensitivity analysis to assess the impact on the overall treatment effects of inclusion of trials which do not report an intention-to-treat analysis, have high rates of participant attrition, or have other missing data.

Each outcome will be combined and calculated using the statistical software RevMan 5.1, according to the statistical guidelines referenced in the current version of the *Cochrane Handbook for Systematic Reviews of Interventions* [12]. The Mantel-Haenszel method will be used for the fixed effects model if tests of heterogeneity are not significant. If statistical heterogeneity is observed (*I* ^2^ ≥ 50% or *P* < 0.1), the random effects model will be chosen. If heterogeneity is substantial, a systematic narrative synthesis will be provided with information presented in the text and tables to summarize and explain the characteristics and findings of the included studies. The narrative synthesis will explore the relationship and findings both within and between the included studies, in line with the guidance from the Centre for Reviews and Dissemination.

### Meta-bias

In order to determine whether reporting bias is present, we will determine whether the protocol of the RCT was published before recruitment of patients of the study was started. We will evaluate whether selective reporting of outcomes is present (outcome reporting bias). We will compare the fixed effects estimate against the random effects model to assess the possible presence of small-sample bias in the published literature (i.e., in which the intervention effect is more beneficial in smaller studies). In the presence of small-sample bias, the random effects estimate of the intervention is more beneficial than the fixed effects estimate. The potential for reporting bias will be further explored by funnel plots if ≥10 studies are available.

### Reporting

The quality of evidence will be reported according to the Grading of Recommendations Assessment, Development and Evaluation working group methodology (GRADE). The quality of evidence will be assessed across the domains of risk of bias, consistency, directness, precision, and publication bias. This will include the impact of any ongoing registered clinical trial on the results of our review. Quality will be adjudicated as high (further research is very unlikely to change our confidence in the estimate of effect), moderate (further research is likely to have an important impact on our confidence in the estimate of effect and may change the estimate), low (further research is very likely to have an important impact on our confidence in the estimate of effect and is likely to change the estimate), or very low (very uncertain about the estimate of effect). Furthermore, we will discuss the general impact and implications of the review findings on the current trend and guidelines for cleft palate repair management.

### Registration

Our systematic review protocol has not been registered with the International Prospective Register of Systematic Reviews (PROSPERO).

## Discussion

Evidence regarding the optimal surgical technique and timing of cleft palate surgery is lacking, with no gold standard management to date [2]. A critical appreciation of how complication rates differ between different surgical procedures and with different time points of repair is critical to optimize the management of these patients. The methodology of this systematic review follows the Preferred Reporting Items for Systematic Reviews and Meta-Analysis Protocols (PRISMA-P) guideline in order to strengthen the comprehensiveness, reliability, and quality of the review [13]. It allows for greater transparency and offers the ability to compare protocols between different systematic reviews. Additional file 2 shows the PRISMA-P guideline used to create the protocol. Knowledge gained from this systematic review will be disseminated through presentation at conferences and publication in a peer-reviewed journal. The objective of this review will be to provide the cleft surgeon with an evidence-based approach for cleft palate surgery, with the end goal of improving patient care.

## Additional files

Additional file 1: Search strategy. Search strategy used to generate review articles. (DOCX 18 kb)
Additional file 2: PRISMA-P checklist. PRISMA-P checklist guideline used to create protocol for this systematic review. (DOCX 28 kb)

## Abbreviations

IVVP: Intravelar veloplasty
PRISMA-P: Preferred Reporting Items for Systematic Reviews and Meta-Analysis Protocols
RCT: Randomized controlled trials
VPI: Velopharyngeal insufficiency
