# Supplementary material for: Post-operative outcomes after cleft palate repair in syndromic and non-syndromic children: a systematic review protocol
Source: Syst Rev. 2017 Mar 9;6:52. doi: 10.1186/s13643-017-0438-2 (PMC5345151; doi:10.1186/s13643-017-0438-2)
Supplement: Additional file 1: — Search strategy. Search strategy used to generate review articles. (DOCX 18 kb) [file 13643_2017_438_MOESM1_ESM.docx]

**Additional file 1**

**Search Strategy**

Description: Search strategy used to generate review articles

Database: Embase Classic+Embase <1947 to 2016 May 05>, Ovid MEDLINE(R) In-Process & Other Non-Indexed Citations and Ovid MEDLINE(R) <1946 to Present>

Search Strategy:

--------------------------------------------------------------------------------

1     cleft palate/su (11955)

2     soft palate/su (1854)

3     hard palate/su (790)

4     palatoplasty/ (1542)

5     (veloplasty or palatoplasty).tw. (1753)

6     (palat* adj3 (repair or reconstruct* or surgery or treatment)).tw. (5986)

7     (furlow adj3 (double or Z-plasty)).tw. (125)

8     von langenbeck.tw. (272)

9     (langenbeck adj3 (repair or reconstruct* or surgery or approach or technique)).tw. (328)

10     veau-wardill-kilner.tw. (62)

11     vomer flap*.tw. (126)

12     or/1-11 (18487)

13     oral fistula/ (692)

14     fistul*.tw. (205814)

15     palatopharyngeal incompetence/ (3546)

16     (velopharyngeal adj3 (dysfunction or function or incompetence or insufficiency or inadequacy)).tw. (3292)

17     ((facial growth or midfac* growth or mandibular or maxilla* or cephalometric) adj3 (deformit* or outcome* or measurement* or hypoplasia or retardation or analysis or growth)).tw. (16247)

18     (midfac* adj3 (hypoplasia or deformit*)).tw. (1405)

19     face growth/ (882)

20     growth stud*.tw. (4633)

21     "Velopharyngeal Insufficiency"/ (2837)

22     or/13-21 (232527)

23     12 and 22 (3903)

24     case report/ not review/ (3748136)

25     23 not 24 (3478)

26     25 use emczd (1753)

27     Cleft Palate/su (11955)

28     Palate, Soft/su (1854)

29     Palate, Hard/su (790)

30     Reconstructive Surgical Procedures/ and Cleft Palate/ (1726)

31     (palat* adj3 (repair or reconstruct* or surgery or treatment)).tw. (5986)

32     (veloplasty or palatoplasty).tw. (1753)

33     (furlow adj3 (double or Z-plasty)).tw. (125)

34     von langenbeck.tw. (272)

35     (langenbeck adj3 (repair or reconstruct* or surgery or approach or technique)).tw. (328)

36     veau-wardill-kilner.tw. (62)

37     vomer flap*.tw. (126)

38     or/27-37 (18356)

39     Oral Fistula/ (692)

40     Velopharyngeal Insufficiency/ (2837)

41     (velopharyng* adj3 (insufficienc* or incompetence or inadequacy or flap or dysfunction or function)).tw. (3333)

42     ((facial growth or midfac* growth or mandibular or maxilla* or cephalometric) adj3 (deformit* or outcome* or measurement* or hypoplasia or retardation or analysis or growth)).tw. (16247)

43     growth stud*.tw. (4633)

44     face/ab (5057)

45     hypoplasia.mp. (60158)

46     fistula.tw. (151861)

47     (midface adj3 deformit*).tw. (70)

48     hypoplasia.tw. (49440)

49     or/39-48 (238799)

50     38 and 49 (3734)

51     (case reports not review).pt. (1671899)

52     50 not 51 (3496)

53     52 use prmz (1698)

54     26 or 53 (3451)

55     remove duplicates from 54 (2289)

56     55 use prmz (1678)

57     55 use emczd (611)

***************************

Database: EBM Reviews - Cochrane Central Register of Controlled Trials <April 2016>

Search Strategy:

--------------------------------------------------------------------------------

1     Cleft Palate/su (139)

2     Palate, Soft/su (6)

3     Palate, Hard/su (0)

4     Reconstructive Surgical Procedures/ and Cleft Palate/ (18)

5     (palat* adj3 (repair or reconstruct* or surgery or treatment)).tw,kw. (233)

6     (veloplasty or palatoplasty).tw,kw. (61)

7     (furlow adj3 (double or Z-plasty)).tw,kw. (3)

8     von langenbeck.tw,kw. (9)

9     (langenbeck adj3 (repair or reconstruct* or surgery or approach or technique)).tw,kw. (12)

10     veau-wardill-kilner.tw,kw. (0)

11     vomer flap*.tw,kw. (2)

12     or/1-11 (340)

13     Oral Fistula/ (6)

14     Velopharyngeal Insufficiency/ (23)

15     (velopharyng* adj3 (insufficienc* or incompetence or inadequacy or flap or dysfunction or function)).tw,kw. (43)

16     ((facial growth or midfac* growth or mandibular or maxilla* or cephalometric) adj3 (deformit* or outcome* or measurement* or hypoplasia or retardation or analysis or growth)).tw,kw. (319)

17     growth stud*.tw,kw. (86)

18     face/ab (0)

19     hypoplasia.tw,kw. (126)

20     fistula.tw,kw. (1259)

21     (midface adj3 deformit*).tw,kw. (0)

22     or/13-21 (1806)

23     12 and 22 (63)

Pubmed  - May 06, 2016

(((((((((((((((((((palatoplasty[Title/Abstract]) OR veloplasty[Title/Abstract]) OR (palate[Title/Abstract] AND surgery[Title/Abstract])) OR (palate[Title/Abstract] AND repair[Title/Abstract])) OR (palate[Title/Abstract] AND reconstruction[Title/Abstract])) OR (double AND furlow)) OR z plasty) OR von langenbeck) OR (langenbeck AND repair)) OR (langenbeck AND surgery)) OR (langenbeck AND reconstruction)) OR veau-wardill-kilner) OR vomer flap)) AND (((((((((fistula[Title/Abstract]) OR velopharyngeal dysfunction[Title/Abstract]) OR velopharyngeal incompetence[Title/Abstract]) OR velopharyngeal insufficiency[Title/Abstract]) OR velopharyngeal inadequacy[Title/Abstract])

OR deformity[Title/Abstract]) OR hypoplasia[Title/Abstract]) OR growth[Title/Abstract]) OR palatopharyngeal incompetence[Title/Abstract]))))) AND ((pubstatusaheadofprint OR publisher[sb] OR pubmednotmedline[sb]))) 166
