# Supplementary material for: Post-operative outcomes after cleft palate repair in syndromic and non-syndromic children: a systematic review protocol
Source: Syst Rev. 2017 Mar 9;6:52. doi: 10.1186/s13643-017-0438-2 (PMC5345151; doi:10.1186/s13643-017-0438-2)
Supplement: Additional file 2: — PRISMA-P checklist. PRISMA-P checklist guideline used to create protocol for this systematic review. (DOCX 28 kb) [file 13643_2017_438_MOESM2_ESM.docx]

**Additional file 2**

**PRISMA-P checklist**

Description: PRISMA-P checklist guideline used to create protocol for this systematic review

**PRISMA-P (Preferred Reporting Items for Systematic review and Meta-Analysis Protocols) 2015 checklist: recommended items to address in a systematic review protocol***

| **Section and topic Item No** | | **Checklist item** |
| --- | --- | --- |
| **ADMINISTRATIVE INFORMATION** | |  |
| Title:  Identification | 1a | Post-operative outcomes of various cleft palate repair methods in syndromic and non-syndromic children: protocol for a meta-analysis |
| Registration | 2 | If registered, provide the name of the registry (such as PROSPERO) and registration number  Our systematic review protocol was not registered with the International Prospective Register of Systematic Reviews (PROSPERO). |
| Authors: Contact | 3a | Provide name, institutional affiliation, e-mail address of all protocol authors; provide physical mailing address of corresponding author  Corresponding author: Zach Zhang^1^ (zzhan163@uottawa.ca)  Michael Stein, MD^2^ (micstein@toh.ca)  Claudia Malic, MD MRCS FRCS (Plast)^2^ (cmalic@cheo.on.ca)  Nigel Mercer, MBBCh, FRCS(Plast) ^3^ (nmercer1@me.com)  ^1^Faculty of Medicine, University of Ottawa, Ottawa, Canada  ^2^Division of Plastic Surgery, Department of Surgery, University of Ottawa, Ottawa, Canada  ^3^ Plastic Surgery Department, Bristol NHS Trust, Bristol, UK  Mailing address: 221 Station Boulevard, Unit 7, Ottawa, ON, Canada, K1G 4C7 |
| Contributions | 3b | Describe contributions of protocol authors and identify the guarantor of the review  Dr. NM contributed to the project idea and editing of manuscript. Dr. CM is the guarantor and also contributed to project idea, study design, data interpretation, supervision of manuscript writing, and revision. Dr. CM and Dr. NM provided expertise on various cleft palate repair methods and outcome complications. All authors contributed to the development of the selection criteria, the risk of bias assessment strategy, and data extraction criteria. ZZ and Dr. MS drafted the manuscript. ZZ developed the search strategy with help from Dr. MS. ZZ and Dr. CM will contribute towards reviewing the articles. All authors read, provided feedback, and approved the final manuscript. |
| Amendments | 4 | If the protocol represents an amendment of a previously completed or published protocol, identify as such and list changes; otherwise, state plan for documenting important protocol amendments  In the event of protocol amendments, the date of each amendment will be accompanied by a description of the change and the rationale |
| Support: Sources | 5a | Indicate sources of financial or other support for the review  This meta-analysis has no financial support. |
| Sponsor | 5b | Provide name for the review funder and/or sponsor  The sponsors will be Dr. Claudia Malic and Dr. Nigel Mercer along with European Cleft Organisation – a European based charity which promotes the excellence in the care of patients with cleft pathology. |
| Role of sponsor or funder | 5c | Describe roles of funder(s), sponsor(s), and/or institution(s), if any, in developing the protocol  The sponsors will play an active role in designing the study and the PRISMA protocol and will be the assessors in extracting the data from the literature search. |
| **INTRODUCTION** |  |  |
| Rationale | 6 | Describe the rationale for the review in the context of what is already known  Please see Background.  Please see Background. |
| Objectives | 7 | Provide an explicit statement of the question(s) the review will address with reference to **participants, interventions, comparators,** and outcomes (PICO)  The aim for our meta-analysis is to evaluate the effectiveness and complications of cleft palate repair in syndromic and non-syndromic children. To this end, the proposed meta-analysis will answer the following questions:   1. What are the comparative effectiveness and complication rates of various cleft palate repair techniques, namely intravelar veloplasty compared to Furlow double-opposing Z-plasty for repair of the soft palate and comparisons between Von Langenbeck palate repair, Veau-Wardill-Kilner palatoplasty, Bardach two-flap palatoplasty, pushback palate repair, hybrid palatoplasty and vomer flap for repair of the cleft palate? 2. Does the timing of the cleft repair in a child’s facial growth with the possibility of performing orthognatic surgery when the patients attain the skeletal maturity? 3. For question 1 and 2 above, the complications reviewed are the rate of palatal fistula formation, velopharyngeal insufficiency, and midface growth retardation. |
| **METHODS** |  |  |
| Eligibility criteria | 8 | Specify the study characteristics (such as PICO, study design, setting, time frame) and report characteristics (such as years considered, language, publication status) to be used as criteria for eligibility for the review  Studies will be selected according to the criteria outlined below.  Please see methods. |
|  | 9 | Describe all intended information sources (such as electronic databases, contact with study authors, trial registers or other grey literature sources) with planned dates of coverage  Please see Methods – Search strategy. |
| Search strategy | 10 | Present draft of search strategy to be used for at least one electronic database, including planned limits, such that it could be repeated  Please see Additional File 1. |
| Study records: Data management | 11a | Describe the mechanism(s) that will be used to manage records and data throughout the review  Please see Methods – Screening. |
| Selection process | 11b | State the process that will be used for selecting studies (such as two independent reviewers) through each phase of the review (that is, screening, eligibility and inclusion in meta-analysis)  Please see Methods – Screening. |
| Data collection process | 11c | Describe planned method of extracting data from reports (such as piloting forms, done independently, in duplicate), any processes for obtaining and confirming data from investigators  Please see Methods – Data extraction. |
| Data items | 12 | List and define all variables for which data will be sought (such as PICO items, funding sources), any pre-planned data assumptions and simplifications  Please see Methods – Data extraction. |
| Outcomes and prioritization | 13 | List and define all outcomes for which data will be sought, including prioritization of main and additional outcomes, with rationale  The primary outcome id the rate of complication from the cleft palate repair, namely the rate of palatal fistula, velopharyngeal insufficiency, and midface hypoplasia. |
| Risk of bias in individual studies | 14 | Describe anticipated methods for assessing risk of bias of individual studies, including whether this will be done at the outcome or study level, or both; state how this information will be used in data synthesis  Please see Methods – Data analysis. |
| Data synthesis | 15a | Describe criteria under which study data will be quantitatively synthesised  Please see Methods – Data synthesis. |
|  | 15b | If data are appropriate for quantitative synthesis, describe planned summary measures, methods of handling data and methods of combining data from studies, including any planned exploration of consistency (such as I^2^, Kendall’s τ)  Please see Methods – Data synthesis. |
|  | 15c | Describe any proposed additional analyses (such as sensitivity or subgroup analyses, meta-regression)  Please see Methods – Data synthesis. |
|  | 15d | If quantitative synthesis is not appropriate, describe the type of summary planned  Please see Methods – Data synthesis. |
| Meta-bias(es) | 16 | Specify any planned assessment of meta-bias(es) (such as publication bias across studies, selective reporting within studies)  Please see Methods – Meta-bias. |
| Confidence in cumulative evidence | 17 | Describe how the strength of the body of evidence will be assessed (such as GRADE)  Please see Methods – Reporting. |

*** It is strongly recommended that this checklist be read in conjunction with the PRISMA-P Explanation and Elaboration (cite when available) for important clarification on the items. Amendments to a review protocol should be tracked and dated. The copyright for PRISMA-P (including checklist) is held by the PRISMA-P Group and is distributed under a Creative Commons Attribution Licence 4.0.**
